# The Psychic Values of Light, Shade, Form and Color

**Published:** 1914-01

**Authors:** 


THE PSYCHIC VALUES OF LIGHT, SHADE, FORM AND COLOR.—F. Park Lewis, M. D., Buffalo, *Trans. of Illuminating Engineering Society*, Oct., 1913. In its last analysis the physics of light must be considered in the effect of shade, form and color upon the human emotions, feelings and sensations. No single factor more definitely dominates the lives of men than the impressions made upon them by what they see. The dignity and beauty of our surroundings inspire to higher living and better citizenship. We are brought in relationship to the external world only through our special senses. Were all the other senses abolished while the intelligence remained, there would be no possibility of communication with the outside world. Light and color are not the waves of different amplitudes in the ether but are the results of the impressions produced upon our consciousness by these external influences. We perceive objects only by reason of the reflected light from their surfaces. Could we imagine a condition in which surfaces would reflect no light, objects could not be seen even though light were present. The element of beauty has not only an esthetic value; there are by-products, if they may be so called, which are incidentally developed and which have even a greater bearing upon our lives. In a mining district in Pennsylvania the workmen were encouraged by competitive prizes to make beautiful gardens in back yards which had been filled with debris. The effect was not only to beautify the district and to give an agreeable change of occupation with physical betterment, but an increase of civic interest. The mental and moral effect of light and shade cannot be ignored. Excessive lighting brings out sordid details with unpleasant glare which is as bad in effect as insufficient light. The beauty of light sources should not be forgotten. Good lights for the poor will make the home beautiful. Collaboration is urged on the part of illuminating engineers, architects, school men, ophthalmologists, and others to secure broader instruction on the care of the eyes.

